# Ixazomib-lenalidomid-dexamethasone (IRd) in relapsed refractory multiple myeloma (RRMM)—multicenter real-world analysis from Germany and comparative review of the literature

**DOI:** 10.1007/s00277-025-06441-8

**Published:** 2025-06-05

**Authors:** Hanna Löffler, Magdalena Braun, Heike Reinhardt, Amelie Rösner, Gabriele Ihorst, Jan Räder, Sina Wenger, Annamaria Brioli, Marie von Lilienfeld-Toal, Maika Klaiber-Hakimi, Eva-Marie Fehr, Markus Munder, Katharina Epp, Karolin Trautmann-Grill, Sarah Decker, Holger Hoff, Ralph Wäsch, Monika Engelhardt

**Affiliations:** 1 Department of Hematology and Oncology, Faculty of Freiburg, University of Freiburg Germany, Freiburg, Germany; 2 Comprehensive Cancer Center Freiburg, Freiburg, Germany; 3 Clinical Trial Center Clinical Trials Unit, Faculty of Medicine, University of Freiburg, University of Freiburg, Freiburg, Germany; 4 Klinik Und Poliklinik Für Innere Medizin C, Hämatologie Und Onkologie, Universitätsmedizin Greifswald, Greifswald, Germany; 5 Klinik Für Innere Medizin II, Universitätsklinikum Jena, Comprehensive Cancer Center Central Germany, Campus Jena, Jena, Germany; 6 Klinik Für Hämatologie, Hämostaseologie, Onkologie Und Stammzelltransplantation, Medizinische Hochschule Hannover, Hannover, Germany; 7 Institut Für Diversitätsmedizin, Ruhr-Universität Bochum, Bochum, Germany; 8 Klinik Für Hämatologie, Onkologie Und Palliativmedizin, Marien Hospital Düsseldorf, Düsseldorf, Germany; 9 Department of Hematology and Medical Oncology, University Medical Centre of the Johannes Gutenberg University Mainz, Mainz, Germany; 10 Department of Hematology and Oncology, Diakonissen-Stiftungs-Krankenhaus Speyer, Speyer, Germany; 11 University Hospital Dresden, Dresden, Germany; 12 Takeda Pharma Vertrieb GmbH & Co. KG, Berlin, Germany

**Keywords:** Multiple Myeloma (MM), Relapsed/refractory (RR), Ixazomib-lenalidomide-dexamethasone (IRd), Real Word Evidence (RWE), Real world data (RWD), German RRMM patients

## Abstract

With the introduction of novel agents (proteasome inhibitors (PIs), immunomodulatory drugs (IMiDs), immuntherapeutics), the outcome of multiple myeloma (MM) patients has improved. The oral PI ixazomib was authorized in the EU in 11/2016 and is indicated in combination with lenalidomide and dexamethasone (IRd) for the treatment of patients with relapsed/refractory MM (RRMM). The study aim was to identify the typical real world (RW) RRMM patient population in Germany treated with IRd and to compare the outcome to prior IRd cohorts. Our retrospective study included consecutive RRMM patients receiving IRd in six large German hospitals between 06/2017 and 12/2021. We identified 24 consecutive RRMM patients within these six institutions. Their median age was 68 years at the start of IRd; 21% carrying high-risk cytogenetics [del17p, t(4;14) and/or t(14;16)]. Patients had a median of 2 prior treatment lines (range, 1–5), prior PI/IMiD exposure was 100%/58%, comparable to previous real world evidence (RWE)-studies. With a median follow-up of 37.7 months, the overall response rate was 70.8%, and median PFS and OS were 22.0 and 62.2 months, respectively. With these German data, our study is closing an important gap of IRd real world data (RWD) reporting across Europe, providing median PFS/OS data and comparison to prior IRd studies. IRd is a well-tolerated and effective triplet regimen, often given as 2nd or 3rd-line therapy in RRMM, with results confirming the registration trial and other international RW-analyses.

**Supplementary Information:**

The online version contains supplementary material available at 10.1007/s00277-025-06441-8.

## Introduction

The treatment of multiple myeloma (MM) has evolved considerably in recent decades. New therapies, such as proteasome inhibitors (PI) or immunomodulating therapies (IMiDs) like lenalidomide (R), have significantly improved the outcome for patients [1]. Due to the large number of new treatment options today, not only the efficacy and tolerability data from randomized controlled trials (RCTs) play a role in the therapy selection, rather, data obtained from observational studies are also valuable [2–5]. In addition to the biology of the disease and the fitness of the patient [6, 7] the preferences of patients need to be considered. These patient-centered approaches may also include patient advocacy groups that are involved in educating, communicating, and planning clinical trials and answering essential questions to meet patient demands and thereby further improve future interventions, clinical trials and standard of care treatment options [8–10].

Ixazomib (I) is a reversible, boron-containing PI that is available orally as the prodrug ixazomib citrate. It particularly inhibits the chymotrypsin-like activity of the beta5 subunit of the 20S proteasome. In the EU, ixazomib is approved since November 2016 in combination with lenalidomide and dexamethasone (d; IRd) for the treatment of MM in adult patients who have already received prior therapy [11–13]. The efficacy of IRd in MM was evaluated in the TOURMALINE-MM1 clinical trial [14]. This phase 3 trial was a randomized, double-blind, international multicenter study. It compared the combination of IRd with placebo, lenalidomide and dexamethasone (Pbo-Rd) in MM patients with 1–3 prior lines of therapy. Treatment was given in a 28-day cycle, with ixazomib given on days 1, 8 and 15, lenalidomide on days 1 to 21, and dexamethasone on days 1, 8, 15 and 22. The primary endpoint of the study was progression-free survival (PFS) [14]. After a median follow-up of approximately 15 months, treatment with IRd resulted in a 6-month increase in PFS compared to Pbo-Rd with 20.6 months vs 14.7 months, respectively [14] Notably, the side effect profile of IRd was similar to Pbo-Rd. The most common adverse reactions were hematological events, gastrointestinal events and rash. Importantly, the addition of ixazomib to Rd did not adversely affect patients'reported quality of life (QoL) [14, 15].

Numerous real-world studies (RWE) have confirmed the efficacy, safety and convenience of ixazomib-based therapies in relapsed/refractory MM (RRMM) and have observed no new safety signals, but equipotent efficacy as compared to TOURMALINE-MM1 [2–5, 16–23]. Whilst the numbers of these RWE are increasing, including those on IRd in RRMM patients, evaluations on the effectiveness and safety of IRd in clinical practice in Germany are lacking.

To close this gap, we conducted this study and examined the characteristics of the typical patient population treated with IRd and the outcomes that can be achieved with IRd under real-world conditions at six large comprehensive cancer centers (CCCs) and compared our data to TOURMALINE-MM1 and prior RWE.

## Methods

**Fig. 1:**
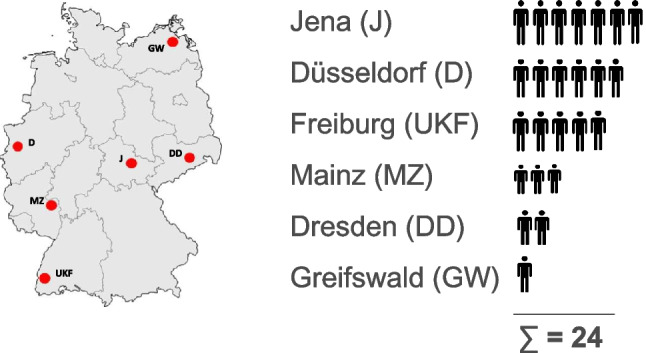
Participating centers and their number of patients

We analyzed data of 24 consecutive RRMM patients within six large CCCs in Germany, which were all, except one (CCC Düsseldorf), university hospitals. Participating centers were the medical centers of Jena, Düsseldorf, Freiburg (UKF), Mainz, Dresden and Greifswald, with IRd patients being included with 7, 6, 5, 3, 2 and 1, respectively (Fig. 1). All of the included patients had symptomatic RRMM (ICD-10 C90.0; MM: in need of systemic combination treatment), and were treated with IRd in accordance with the IRd-approval after at least one prior therapy. Excluded were IRd patients treated within a clinical trial or not in accordance with the IRd-market authorization (Supplementary Table 1). Patients started IRd treatment between June 2017 and December 2021.

**Fig. 2:**
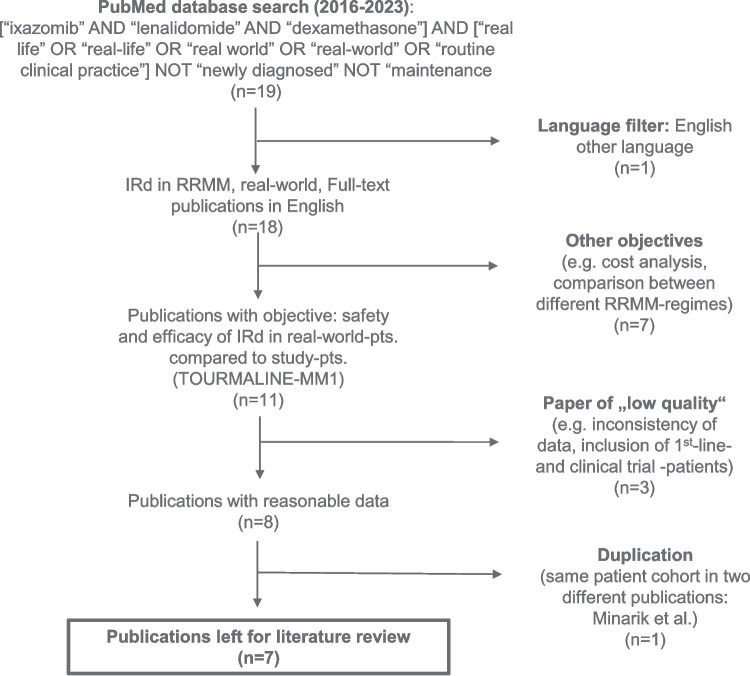
Flow diagram of performed literature search

We also performed a literature review (PubMed search 2016–2023) of IRd use in RWE settings from the Tourmaline-MM1 study start 2012 until 12/2021). Seven relevant papers were identified via a PubMed database search (Fig. 2). We compared key parameters of the TOURMALINE-MM1 register trial with the RWE from these publications and our own data, as summarized in Tables 1, 2, 3 and 4. The search criteria for the PubMed database search are shown in Fig. 2. The search terms were"ixazomib"AND"lenalidomide"AND"dexamethasone"in addition to"real life"OR"real-life"OR"real world"OR"real-world"OR"routine clinical practice"and excluding"newly diagnosed"or"maintenance". This search yielded 19 literature hits in PubMed, of which one publication was not in English and was not considered. Due to different objectives in some studies, such as cost analyses or comparisons between other RRMM treatments, seven publications were excluded. Three publications were excluded due to low quality and one due to duplication in this literature search. In the end, seven publications were included in the further evaluation and used for comparison with our own data (Fig. 2).

**Table 1:** Patient characteristics (*n = *24)

	Median (range)	*n* (%)
Age at start of IRd treatment (years)	68 (43–87)	
Male/female patients		13 (54.2)/11 (45.8)
IgG/IgA/LC-only	14/5/5	
Kappa/lambda LC type	14/10	
ISS: I/II vs. III		16 (66.7)/8 (33.3)
R-ISS: I/II vs. III		18 (75.0)/6 (25.0)
Prior lines of therapy	2 (1–5)	
prior ASCT		14 (58.3)
prior PI		24 (100.0)
prior IMiD		14 (58.3)
refractory to PIs		5 (21)
refractory to IMiDs		3 (13)
High-risk/standard-risk cytogenetics		5 (21)/19 (79)
del 17p		2
t(4;14)		2
t(14;16)		1
Median albumin (g/dl; range)	3.3 (2.2–4.2)	
Median beta-2-microglobulin (mg/l; range)	3.5 (1.9–13.1)	
Median LDH (U/l; range)	210 (112–370)	
Median creatinine (mg/dl)	1.1 (0.7–3)	
Time from diagnosis to IRd start (months)	37.6 (14.7–113.0)	
IRd cycles Ixazomib dose reduction: no/yes	12.5 (1–52)	20 (83.3)/4 (16.7)
IRd not discontinued		7 (29.2)
IRd discontinued due to PD		14 (58.3)
IRd discontinued due to patient decision		1 (4.2)
IRd discontinued due to AE		1 (4.2)
IRd discontinued due to other reason		1 (4.2)
Best IRd response:		
ORR (CR, VGPR, PR)		17 (70.8)
Clinical benefit rate (CR, VGPR, PR, MR, SD)		24 (100)
CR		1 (4.2)
VGPR/PR		12 (50.0)/4 (16.7)
MR/SD		5 (20.8)/2 (8.3)
PD		0 (0)
Patients alive/dead		15 (62.5)/9 (37.5)
Median PFS (range)	22.0 (1.7–62.2)	
Median OS (range)	62.2 (2.4–62.2)	
Median follow-up (months)	37.7	

Abbreviations and definitions: *IRd* Ixazomib-lenalidomide-dexamethasone, *LC-only* light chain secreting MM alone, *ISS* international staging system; R-ISS: revised ISS, *ASCT* autologous stem cell transplantation, *PI* proteasome inhibitor, *IMiD* immunomodulatory drug, high-risk/standard-risk cytogenetics: according to IMWG, *LDH* lactate dehydrogenase, *PD* disease progression, *AE* adverse events, *ORR*overall response rate, *CR* complete remission, *VGPR* very good partial remission, *PR* partial remission, *MR* minimal response, *SD* stable disease, *PFS* progression free survival, *OS* overall survival

**Table 2:** Incidence of drug related adverse events under therapy with IRd (*n = *24)

Adverse event	Incidence rate (%)
Diarrhoea	17%
Cytopenia	13%
Respiratory infection	8%
Rash/skin infection	4%
Muscle cramps	4%

Abbreviations and definitions: *IRd* Ixazomib-lenalidomide-dexamethasone

**Table 3:** Comparison of our data on IRd, to the approval study (Tourmaline -MM1) and other selected publications (Pubmed literature search: 2016–2023)

# of trials	Study name or first author	Study design	Country participating	# of patients (IRd cohort)	Median age (range)	Median # of prior therapies (range)	Prior exposure to -PI -IMiD [*n* (%)]	Dose reduction of Ixa due to AEs [*n* (%)]	IRd discontinuation due to -PD -AEs [*n* (%)]	Median PFS/OS (months)
1	Tourmaline-MM1 2016	RCT, placebo-controlled: IRd vs. Rd	26 countries	360	66 (38–91)	1 (1–3)	249 (69) 193 (54)	N/A	124 (34) 60 (17)	20.6/54
2	**Our data 2024**	**Retrospective**	**Germany, 6 sites**	**24**	**68 (43–87)**	**2 (1–5)**	**24 (100)** **14 (58)**	**4 (16.7)**	**14 (58)** **1 (4.2)**	**22.0/62.2**
3	Lee et al. 2023	Retrospective	Korea	60	68 (53–82)	1 (1–2)	58 (97) 29 (48)	27 (45)	20 (33) 22 (37)	25.9/n.r
4	Minarik et al. 2021 + 2022	Prospective, head-to-head (IRd vs. Rd)	Czech Republic	127	66 (41–84)	1 (1–9)	123 (97) 64 (50)	N/A	38 (30) 8 (7)	17.5/40.9
5	Sokol et al. 2022	Retrospective	Slovakia	106	63 (44–78)	2 (1–7)	101 (95) 51 (48)	8 (7.5)	56 (56) 3 (3)	43/n.r
6	Ziff et al. 2021	Retrospective	UK	85	65 (32–84)	2 (1–4)	85 (100) 52 (61.2)*	20 (23.5)	35 (41.2) 11 (12.9)	n.r
7	Maouche et al. 2020	Retrospective	UK	116	68.5 (32–86)	2 (1–4)	111 (95.7) 92 (79.3)	8 (7)	35 (30) 6 (5)	n.r
8	Varga et al. 2019	Retrospective	Hungary	77	66 (N/A)	2 (1–3)	76 (99) 77 (99)	9 (11)	N/A 5 (6.5)	N/A
9	Terpos et al. 2020	Retrospective	Greece, UK, Czech Republic	155	68.1 (40–85)	1 (1–7)	150 (97) 86 (56)	N/A	48 (31) 14 (9)	N/A

Abbreviations and definitions: * At least (IMiDs in general not mentioned, but Thalidomide in *n = *52), #: number, *AEs* adverse events, *IMiD* immunomodulatory drug, *IRd* ixazomib/lenalidomide/dexamethasone, Ixa: ixazomib, *OS* overall survival, *PD* progressive disease, *PI* proteasome inhibitor, *PFS* progression free survival, *RCT* randomized controlled clinical trial, *N/A* not applicable, because not given/available in publication; *n.r.* not reached

**Table 4:** Differences and additions with current analysis to IRd knowledge of most recent IRd real world (RWE) analyses

#	First author	Reference	Key findings	Weakness/limitations	Additions with this analysis
1	Löffler H	Current analysis 2025	21% HR CG, PI/IMID exposure 100%/58%, Long median FU: 38 ms, ORR 71%, CBR 100%, PFS/OS: 22/62 ms Excellent therapy tolerance as outpt therapy, Low therapy discontinuation rate due to AEs (4%) Pts alive 63% RWE analysis from 6 large German CCCs with no prior German RWE data	Retrospective RWE study, Limited number of pts, but with more pts not adding to the usefulness of the data in current IO treatment scenarios	First multicenter German RWE study ChemoCompile IRd protocol generated for external use [24] Longest follow-up, Lowest dose reduction and discontinuation rate, Best IRd tolerance, possibly due to ChemoCompile protocol recommendations, [24] Most favorable PFS and OS rates No PNP aggravation/signal
2	Terpos E	AoH 2020 [2]	RWE in Greece, UK + Czech Republic	RWE from different countries with much divergent health care and support systems Median prior line befor IRd: only 1 Median IRd exposure: fairly short with 9.6 ms IRd treatment assessment ≥ 6 ms without Landmark analysis performed [33] PNP rate substantial: 35%	
3	Davies F	AoH 2021 [3]	RWE via US electronic health record database, comparing VRd, KRd, DRd, IRd	Retrospective RWE from US electronic health record database, thus data being incomplete, lacking or non-intended for regimen-comparisons, > 17 different regimens being compared (see Fig. 1 [3])	
4	Chari A	Exp Review Hematol 2020 [26]	US RWE comparison of IRd, KRd, VRd	Incomplete US RWE data with different RRMM regimens in diferent lines of treatment being applied with less well-comparable MM settings Retrospectively assessed frailty status that was used to correct for median TTNT—> suggested IRd, KRd and VRd to be similar, which can only be performed if prospectively compared [34]	
5	Hajek R	Future Oncol 2021 [4]	INSIGHT MM registry data from 13 countries used with short FU (15 ms), PFS of 21 ms and Ixa/Len dose reduction and discontinuation rates of 17/36% and 32/30% due to AEs, respectively	Registry data, short FU and substantial dose reductions and stops, albeit IRd is usually well tolerable, possibly due to incomplete data and 13 countries being included for RWE	
6	Leleu X	Future Oncol 2024 [18]	3 observational studies (INSIGHT, UVEA, REMIX) = double reporting to 5	RWE data extention to prior INSIGHT reporting, median FU 19 ms, median PFS 20 ms, ORR 65% Confirmation that IRd is better in earlier than later lines	

Abbreviations and definitions: *IRd* Ixazomib-lenalidomide-dexamethasone, *RWE* real world evidence, *HR CG* high-risk cytogenetics according to IMWG: del17p, t(4;14), t(14;16), *PI/IMiD* proteasome inhibitor/immunmodulatory drugs, *FU* follow-up, *ms* months, *ORR* overall response rate, *CBR* clinical benefit rate, *PFS* Progression free survival, *OS* overal survival, outpt: outpatient, *AEs* adverse events, *pts* patients, *RWE* real world evidence/real world analysis, *CCCs* Comprehensice Cancer Centers, *IO* immunotherapy, *PNP* polyneuropathy, *US* Unites States, *RRMM* relapsed/refractory multiple myeloma, *TTNT* time to next treatment, *Ixa* ixazomib, *Len* lenalidomide

The aim of this project was to determine the current role of IRd in clinical practice in Germany with focus on patient factors, considering individual MM treatment management issues. Thereby, identifying the typical and optimal RRMM patient population in real life use of IRd within German CCCs and as compared to prior IRd results was the focus of this analysis.

All procedures performed were in accordance with the ethical standards of the institution UKF and national research committee and with the 1964 Helsinki declaration and its later amendments or comparable ethical standards.

Informed consent was obtained from all individual participants included in the study.

## Results

### Patient and MM characteristics

The median age of the 24 patients in our study was 68 years (range: 43–87). Male and female patients were 13 and 11, respectively, and ISS and R-ISS stages I/II vs. III were 16 vs. 8 and 18 vs. 6, respectively (Table 1).

The median number of prior lines of therapy before IRd was two (range: 1–5). All 24 patients were pre-treated with a PI (100%) and 58% were pre-treated with an IMiD, mostly with lenalidomide. Patients refractory to PIs and IMiDs were 5 and 3, respectively (Table 1). High-risk or standard-risk cytogenetics according to IMWG were 5 and 19 patients, respectively, with del17p in 2 patients, t(4;14) in 2 patients and t(14;16) in 1 patient (Table 1). Median albumin before IRd start was 3.3 g/dl (range: 2.2–4.2), beta-2-microglobulin 3.5 mg/l (range: 1.9–13.1), LDH 210 U/L (range: 112–370) and serum creatinine 1.1 mg/dl (0.7–3).

### IRd efficacy and safety results

The median time from initial diagnosis to IRd start was 37.8 months and a median of 12.5 IRd cycles were applied (range: 1–52), in most cases with dose reductions (Table 1). In seven patients, IRd was still ongoing at the time of data analysis. Treatment was discontinued due to progression (PD) in 14 patients and in 1 patient each due to the patient’s decision, AE or other causes (Table 1).

**Fig. 3:**
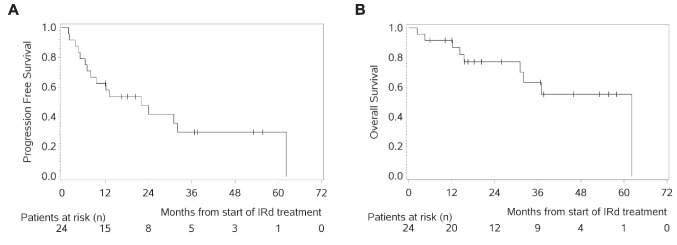
A + B. PFS and OS

Best IRd response with achievement of ≥ CR, VGPR or PR (ORR) was achieved in 17 patients (70.8%) and the clinical benefit rate (CBR: ≥ CR, VGPR, PR, MR, SD) was observed in 24 patients (100%). The median follow-up was 37.7 months, with 15 (62.5%) being still alive, whereas nine patients have deceased due to PD (37.5%). The median PFS (PFS) was 22.0 months (range: 1.7–62.2; Fig. 3A) and the median overall survival (OS) was 62.2 months (range: 2.4–62.2, Fig. 3B; Tables 1).

**Fig. 4:**
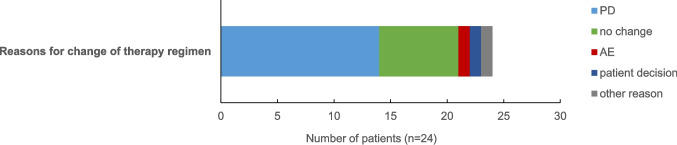
Reasons for change of therapy regimen

Table 2 summarized our AEs: the safety profile was good, AEs were few, generally mild or non-existent and thus the all oral treatment well tolerated (Fig. 4).

### Comparison with pivotal study and existing RWE data

Our German results confirmed the results of prior RWE as summarized in Table 3: the median patient age ranged from 63 to 68.5 years and the median number of prior therapy lines before IRd had been one or two. Most (95%−100%) patients from comparative RWE had been previously treated with a PI and with a prior IMiD in 48%−99%. Dose reductions of ixazomib due to AEs—if this information was reported—were applied in 7%−45% of patients. In addition, therapy was discontinued due to disease progression in 30%−56% and due to AEs in 3%−37% of patients, with most AEs being mild (grade 1–2). Survival data was reported in five of seven studies: the median PFS in other RWE ranged from 17.5 to 43 months. The median OS ranged from 40.9 to not reached (in four studies; Table 3) [2, 16, 19–21].

Therefore, the data collected under RWE conditions was in line with the data from the RCT Tourmaline-MM1 study. In the Tourmaline-MM1 study, the median age of patients was 66 years and median prior therapy lines had been only 1 (range 1–3). Pretreatment with a PI and IMiD had occurred in 69% and 54%, respectively. Therapy with IRd was discontinued due to PD and AEs in 34% and 17% of patients, respectively. Similar to our data, the median PFS and OS in the Tourmaline-MM1 study were 20.6 and 54 months, respectively (Table 3) [15].

Thus, compared to the Tourmaline-MM1 study, patients included in our study received more treatment lines prior to IRd, with a median of 1 vs. 2 prior therapy lines, respectively. Consistently, prior exposure and treatment with PIs and IMiDs was higher in our cohort compared to the Tourmaline-MM1 study with 100% vs. 69% and 58% vs. 54%, respectively (Table 3). Our patients had a higher rate of PD followed by therapy discontinuation compared to Tourmaline-MM1 (58% vs. 34%), however, we observed fewer therapy discontinuations due to AEs (4% vs. 17%, respectively). Premedication with granisetron, cotrim/acyclovir-prophylaxis was sufficient to continue IRd in most patients until progression, as published within the “Das Blaue Buch”, Springer, 8.th edition, 2023 and ChemoCompile, the latter being used throughout Germany, Switzerland and Austria for safe chemotherapy orders and management [24]. The OS in our cohort was 62.2 months, the median PFS 22.0 months, both comparable to the OS/PFS of the Tourmaline-MM1 study (Table 3) [16, 25].

Table 4 summarizes differences and adjuncts with our study to 5 most recent IRd RWE analyses, [2–4, 18, 26]. It outlines the key findings from our analysis and others, including our (and other) limitations, but also notable add-ons, namely our long follow-up, excellent ORR/CBR, PFS/OS and good IRd tolerance, with few side-effects and no PNP aggravation.

## Discussion

The real world evidence (RWE) collected in our multi-center study from Germany provides important insights into the use of the ixazomib, lenalidomide, and dexamethasone (IRd) triplet in relapsed/refractory multiple myeloma (RRMM). Notably, this study is the first RWE analysis of IRd in Germany, offering a valuable contribution to the growing body of evidence on this regimen. Furthermore, our analysis provides a comprehensive comparison with other global RWE studies, helping to contextualize our findings within the broader landscape of IRd treatment (Tables 3 and 4). Our study confirms the findings of others as well as data obtained in the TOURMALINE-MM1 study (summarized in Table 3). However, we also noted clinically relevant differences. For example, the data from our study in Germany, as well as of others, show that in routine clinical practice, patients are more often pre-treated with both PI and IMiDs compared to the RCT TOURMALINE-MM1 study, explaining the higher rate of PD in our study [14]. Nevertheless, our and other RWE studies on the efficacy of the IRd scheme were very comparable to the results of the TOURMALINE-MM1. These results suggest that prior treatment with another PI or IMiD does not necessarily have a negative impact on the efficacy of IRd and that IRd is an effective triplet. In addition, the RWE collected in our study revealed the very good tolerability of the IRd triplet in routine clinical use in Germany. Above all, the low discontinuation rate due to side effects should be emphasized (Tables 1, 2, 3, and 4, Fig. 4). The observed discontinuation rate due to AEs was only 4.2%, which is consistent with the literature and the TOURMALINE-MM1 data (17%) [2, 5, 14, 16, 19–21, 25]. This suggests that IRd is a well-tolerated alternative for patients with RRMM, even for those who have been pretreated with PIs and are seeking an all oral regimen.

Despite our limited patient collective, our results are in line with the recently published data from the INSURE RWE study (Table 4) [18]. A larger patient cohort would be needed to validate our results with greater statistical power and to further understand potential patient subgroup effects. In the INSURE RWE study with > 500 MM patients, it was shown that prior treatment with lenalidomide in non-refractory patients had no negative effect on the efficacy of IRd in a later line of therapy. However, with prior treatment with a PI, shorter PFS and time to next treatment (TTNT) times were observed as compared to PI-naïve patients. Nevertheless, IRd-treated patients may also show rewarding response rates and prolonged therapy benefit. Expectedly, lenalidomide- or PI-refractory patients responded significantly worse to IRd treatment than naïve or non-refractory patients [18].

Of note, since our RWE analysis and the comparison to other global IRd analyses (which we conducted in 2023 comprising 19 studies, as depicted in Fig. 2), as of today (12/2024), 32 RWE IRd-analyses are available, suggesting that experience and knowledge on post-trial and register data are increasing and confirmation of phase 3 trials is essential for the community. Moreover, in times of earlier immunotherapy (IO) agent treatment with bispecific antibodies (BiTEs), chimeric antigen receptor (CAR)-T-cells and antibody drug conjugates (ADCs) being used, patients may become refractory to these novel IO options and are then in need of alternative regimes. Bearing this in mind, IRd is representing a valuable oral treatment option promoting patients’ QoL, albeit formal QoL analyses, as performed previously [27–29] and within the registration trial [15], was not repeated here.

Advantages of our study were that it represents the first IRd RWE analysis from Germany. It also contributes to the existing data from other centers, as summarized in Tables 1, 2, 3, and 4. Comparative analysis of our study results to both TOURMALINE registration study and other RWE analyses has—to the best of our knowledge—not been performed previously (Table 3), add-ons we provide here and that are useful for MM experts, are provided in Table 4. Furthermore, our results highlight the importance of profound knowledge of the IRd-regimen to enable best support of the patients throughout their therapy, securing a low rate of side effects and promoting therapy adherence [24]. Our study has limitations, since the sample size was small, preventing subgroup analyses. However, this population was enrolled as part of a multicenter study incentive, which implied our greatest effort to achieve the largest possible cohort in Germany. For this study, we had inquired all German university and large myeloma centers to contribute to this IRd-experience made in RRMM. Despite our effort, we could not obtain all German centers to participate, but the ones as summarized here, with study protocol, patients’ and ethical support. Since there are numerous efforts of various countries on IRd, worldwide experience does exist and is summarized in our “review of the literature” (Tables 3  and 4). Subgroups had been assessed in larger cohorts, thus was not repeated here. We would argue that IRd has its room in RRMM treatment in those patients, where an all-oral-regimen is preferred, in patients suffering from polyneuropathy (PNP), and those, who prefer to come less frequently to centers for subcutaneous (sc) or intravenous (iv) regimens. Terpos et al. had in their IRd-experience also named high-risk cytogenetics, elderly and/or frail patients and renal impairment as possible features, where this regimen can be considered [2]. In addition, we had patients in Freiburg on VRd, who had or developed PNP, that did subside with IRd being continued without MM progression, since the switch from the PI bortezomib to ixazomib did not increase their PNP symptoms. Of interest, we had a recent International Medical Service patient, seeking a 2.option on 2.line treatment after Daratumumab-Vd pretreatment. This patient is now 87-years old, remained very interested in subsequent treatment, was looking for an all-oral therapy, with a good safety profile and without aggravating his PNP, where indeed IRd had been chosen as his subsequent treatment. This recent patient example further illustrates the above, where IRd can be considered, but without formally including this patient into our study, because his follow-up – different to the other patients – is short.

Additionally, the rapid evolving landscape of anti-myeloma therapy must be considered today. Triplet and quadruplet therapies with PI-/IMiD- and CD38-antibody combinations are currently used in clinical practice, and even upfront IO options with BiTEs and CAR-T-cells are increasingly being explored in clinical trials. As a result, regimens like IRd may become lesser utilized as second- or third-line treatment options, than possibly later, likely since continuous, more intensive and repeated therapy lines are now commonly performed in MM patients [30]. A recent myeloma meeting in Rostock (Saturday, 15.th of March 2025) did indeed show OSHO registry data (https://osho-services.de/services/klinische-studien-und-register) of different PI-, IMID-, CD38-ab-treatment sequences within this registry, demonstrating that IRd was given both upfront (2.−4th line), but also in later lines (≥ 5 th-14.th line; personal communication Profs. Drs. Ch.Junghanss/S.Böttcher). As with any therapy decision in the course of MM, it is essential to consider patients’ comorbidity, family support/personal circumstances, MM-risk profile, prior therapies and preferences. In this regard, one major advantage of Ixazomib, compared to Bortezomib and Carfilzomib, is its oral administration. This is particularly valuable in maintaining patients’ flexibility in daily life, especially for those living far from the nearest hospital or specialized medical practice. For some RRMM patients this is attractive, as they would otherwise face the difficulty of traveling long distances, i.e. twice weekly for Carfilzomib administration, weekly for Bortezomib, likewise for CD38-antibody- and BITE-treatment (at least within the first 8 to longer subsequent weeks). Additionally, since patients receiving Carfilzomib are at higher risk for cardiovascular side effects, the excellent tolerance of Ixazomib is often considered as another advantage, especially for patients with a history of cardiovascular disease (Table 4) [31].

In conclusion, we confirm with consecutive RRMM patients treated within six German CCCs, that the IRd triplet is an effective, safe and all oral treatment option for previously treated patients with symptomatic RRMM, as well have summarized in Table 4, what this study adds to other RWE IRd analyses. Since MM patients value the greater convenience and may prefer oral to injectable medications, all these rare oral triplet options are of interest to MM patients and physicians. For example, all oral treatments are useful for patients who prefer not to travel to clinics or cannot perform self-injection within the community. In addition, oral treatments are less costly for healthcare providers and require less capacity at treatment centers compared to intravenously administered therapies. In consideration of patients preference for oral therapies in more advanced lines of therapy [32] and the fact that IRd is available worldwide today, our RWE study of German patients confirmed that IRd can be considered as a valuable treatment option in RRMM patients.

## Supplementary Information

Below is the link to the electronic supplementary material.

Supplementary file1 (DOCX 31 KB)

Supplementary file2 (PPTX 831 KB)

## Data Availability

No datasets were generated or analysed during the current study.

## Declarations

### Ethcial approval

All procedures performed were in accordance with the ethical standards of the institution UKF and national research committee and with the 1964 Helsinki declaration and its later amendments or comparable ethical standards.
