# Supplementary material for: Ixazomib-lenalidomid-dexamethasone (IRd) in relapsed refractory multiple myeloma (RRMM)—multicenter real-world analysis from Germany and comparative review of the literature
Source: Ann Hematol. 2025 Jun 5;104(7):3713–22. doi: 10.1007/s00277-025-06441-8 (PMC12334530; doi:10.1007/s00277-025-06441-8)
Supplement: Supplementary file 1 — Supplementary file1 (DOCX 31 KB) [file 277_2025_6441_MOESM1_ESM.docx]

**Supplementary Table 1. Inclusion and exclusion criteria for patient accrual to real world evidence (RWE) IRd study**

| **Inclusion criteria** |  |
| --- | --- |
|  | - >18 years |
|  | - RRMM diagnosis according to ICD-10 C90.0 |
|  | - Start IRd between 21.11.2016 – 31.12.2021 |
|  | - Received IRd according to marketing authorisation (≥2^nd^-line treatment) |
| **Exclusion criteria** |  |
|  | - IRd within clinical trial |
|  | - IRd not according to label |
