# Supplementary figures and images for: Ixazomib-lenalidomid-dexamethasone (IRd) in relapsed refractory multiple myeloma (RRMM)—multicenter real-world analysis from Germany and comparative review of the literature
Source: Ann Hematol. 2025 Jun 5;104(7):3713–22. doi: 10.1007/s00277-025-06441-8 (PMC12334530; doi:10.1007/s00277-025-06441-8)

## Slide 1
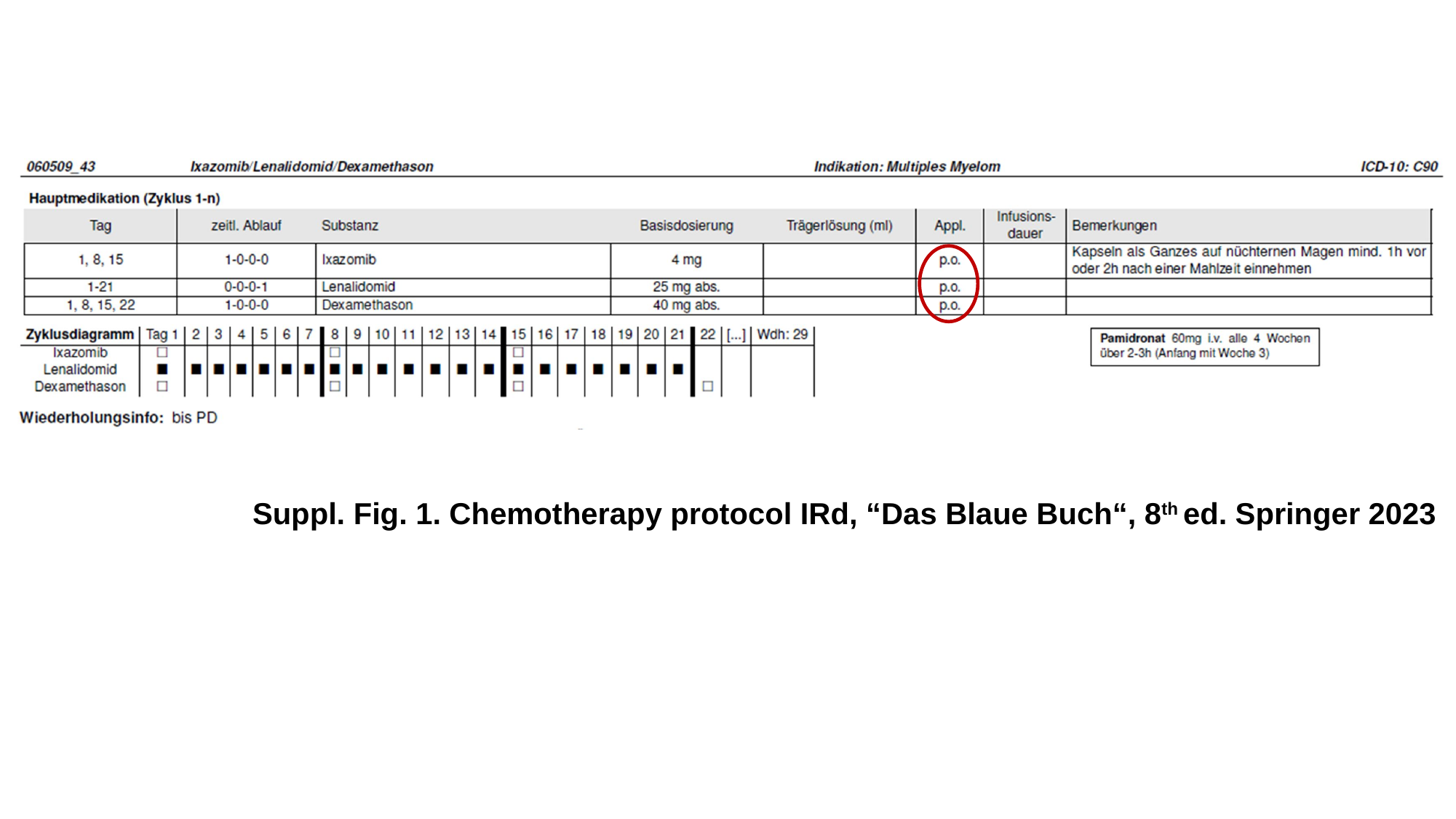

Suppl. Fig. 1. Chemotherapy protocol IRd, “Das Blaue Buch“, 8th ed. Springer 2023

Supplement: Supplementary file 2 — Supplementary file2 (PPTX 831 KB) [file 277_2025_6441_MOESM2_ESM.pptx]
